# Special Issue on “Fruit Metabolism and Metabolomics”

**DOI:** 10.3390/metabo10060230

**Published:** 2020-06-03

**Authors:** Annick Moing, Pierre Pétriacq, Sonia Osorio

**Affiliations:** 1 UMR Biologie du Fruit et Pathologie, Centre INRAE de Nouvelle Aquitaine-Bordeaux, University Bordeaux, INRAE, 71 av Edouard Bourlaux, 33140 Villenave d’Ornon, France; pierre.petriacq@inrae.fr; 2 Bordeaux Metabolome, MetaboHUB, PHENOME-EMPHASIS, Centre INRAE de Nouvelle Aquitaine-Bordeaux, IBVM, 71 av Edouard Bourlaux, 33140 Villenave d’Ornon, France; 3 Department of Molecular Biology and Biochemistry, Instituto de Hortofruticultura Subtropical y Mediterránea “La Mayora”, University of Málaga—Consejo Superior de Investigaciones Científicas (IHSM-UMA-CSIC), 29071 Málaga, Spain; sosorio@uma.es

**Keywords:** fruit metabolomics, developmental metabolomics, stress metabolomics, spatial metabolomics, central metabolism, specialized metabolism, mass spectrometry, nuclear magnetic resonance spectroscopy, omics, multi-omics integration

## Abstract

Over the past 10 years, knowledge about several aspects of fruit metabolism has been greatly improved. Notably, high-throughput metabolomic technologies have allowed quantifying metabolite levels across various biological processes, and identifying the genes that underly fruit development and ripening. This Special Issue is designed to exemplify the current use of metabolomics studies of temperate and tropical fruit for basic research as well as practical applications. It includes articles about different aspects of fruit biochemical phenotyping, fruit metabolism before and after harvest, including primary and specialized metabolisms, and bioactive compounds involved in growth and environmental responses. The effect of genotype, stages of development or fruit tissue on metabolomic profiles and corresponding metabolism regulations are addressed, as well as the combination of other omics with metabolomics for fruit metabolism studies.

The growth and ripening of fruit are multifaceted and highly regulated developmental processes which yield colorful and flavorful tissues for organisms that consume and disperse the seeds therein [1]. Fruits are economically essential and vital for human nutrition and health owing to their content in sugars, organic acids, pigments, volatiles and other nutraceutical compounds [2,3], the metabolisms of which have been widely studied (e.g., [4,5,6]). The shift from single-metabolite analyses to analytical platforms that provide information on hundreds of metabolites has allowed researchers to better describe the links both within metabolites and between metabolism and important agronomic-associated traits. Metabolomics has permitted the identification of changes in the chemical composition of transgenic plants, mutants or populations and has allowed for identifying genomic regions associated with metabolite traits of agronomical value in model fruit species such as tomato and strawberry [7]. Tomato metabolomic studies have been numerous in the past decade [8,9] and have allowed the development of a comprehensive understanding of primary and specialized metabolism pathways and their interplay during fruit growth and development, and in diverse environmental conditions.

The number of teams involved in, and of articles published on, fruit metabolomics has been regularly and progressively increasing. Searching for articles in the Web of Science core collection for metabolomic(s) or metabolome, and fruit in the last decade (Figure 1) revealed an increment from about 20 articles per year in 2010 to about 200 in 2019. The application domains are also increasingly diverse, as illustrated by very recent works ranging, for instance, from the study of the regulation of steroidal glycoalkaloids biosynthesis in Solanaceae [10] to that of the effect of scion/rootstock interaction on the metabolic composition in citrus fruit [11].

This Special Issue focuses on applications of metabolomics within the field of plant sciences for the study of fruit. It contains 12 original research articles and one review article. The contributed research articles are exemplary studies covering primary research applied to fruit species from a basic understanding of metabolism regulation to applications for phenotyping for breeding or defence priming (Figure 2).

This Special Issue covers a range of fruit species, including model fruit (tomato [12,13,14]), temperate (kiwifruit [15], mulberry [16]) or tropical fruit crops (pineapple [17], cashew [18]), genetic resources (melon [19]), and indigenous fruit (Davidson’s plum, finger lime and native pepper berry [20]). Different analytical approaches are covered also: near-infrared spectroscopy (NIRS) [18], gas chromatography coupled to mass spectrometry (GC-MS) [17], liquid chromatography coupled to mass spectrometry (LC-MS) [16,18,21,22], LC-MS/MS [20], and a combination of several analytical strategies including nuclear magnetic resonance spectroscopy (NMR), GC-MS and LC-MS [19] or several omics [15].

The original research articles can be classified according to scientific domains: biochemical phenotyping of genetic resources for collection maintenance or intraspecific classification, generating knowledge about fruit growth, ripening and post-harvest, understanding metabolism, characterizing defence priming, identifying bioactive compounds for human nutrition and health.

Biochemical phenotyping of cashew apple based on LC-MS and near-infrared spectroscopy [18] allowed the identification of a group of accessions, as a proof of concept for their use for the maintenance of a germplasm bank. For melon genetic resources [19], a combination of metabolome approaches enabled an evaluation of metabolomic relationship and its correlation with the genetic genome-by-sequencing distance of melon accessions, revealing that several melon groups, such as Inodorous, grouped in parallel with the genetic classifications, while other genome to metabolome and mineral element associations appeared less clear. A multi-approach study of color mutations introduced in a tomato Italian landrace [12] revealed unexpected biochemical changes. The double mutants expanded the effect on the metabolism of the single mutations by revealing additional additive or epistatic effects that could be of interest for the improvement and diversification of the landrace.

Fruit are complex organs constituted of different tissues. The distribution of metabolites throughout the different fruit tissues, pericarp, placenta, and seeds, was described in chilli pepper [22]. Pericarp showed a higher content of glycosides and terpenoids compared with other parts of the fruit. Placenta was the tissue having the highest content in alkaloids related to capsaicinoids and in tocopherols.

The mechanisms underlying fruit growth during kiwifruit development were investigated using phytohormone profiling with transcriptomics under two carbon supply levels [15]. The results suggest that cytokinins, known to be implicated in cell division, are also involved in fruit cell expansion and growth in kiwifruit. Pineapple ripening was characterized using GC-MS of crown, flesh, and peel samples [17]. Samples could be separated according to ripening phases from early-ripening to late-ripening phases for flesh and peel, which allowed highlighting metabolites correlated to ripening.

Concerning metabolism regulation, a cross-species study using LC-MS was performed to clarify the metabolic regulation of fruit phenolics among three Solanaceae crops, tomato, eggplant and pepper [21]. It allowed identifying the metabolic signatures of phenolics in each species from different fruit tissue-types and ripening stages and provided information for future functional genomics or breeding approaches. The metabolic changes during fruit postharvest storage were reviewed [23], emphasizing the roles that metabolomic platforms can play for better understanding the biochemical bases of postharvest physiology and identifying the numerous, often species-dependent, pathways affected during fruit senescence.

Metabolic correlation networks covering a variety of metabolic traits such as lipophilic and volatile compounds in tomato fruit have shown how the individual metabolic classes are related to yield-related phenotypic traits [13]. Moreover, metabolite-transcript correlation analysis exposed crucial putative genes involved in the biosynthesis of lipids.

Fruit metabolism is tuned in interaction with the abiotic and biotic environments. Immune priming of tomato fruit by the defence inducer ß-aminobutyric acid (BABA) was investigated against three different fruit pathogens using a combination of targeted and untargeted metabolomics [14]. While BABA resulted in a long-lasting induced resistance in tomato fruit against those pathogenic microbes, the primed responses were likely specific to the infection nature, rather than characterizing a common pattern of BABA-induced priming. Further, modelling approaches established that soluble sugars were essential to predict resistance to fruit pathogens.

In relation to human health, in white and black mulberry fruits, combining an LC-MS method with assays for (i) α-glucosidase inhibitory activity and (ii) antioxidant activity [16], allowed for identifying a list of key bioactive compounds with antioxidant and/or α-glucosidase inhibitory activities. Similarly, based on targeted and untargeted metabolite profiling and antioxidant assays on selected Australian native fruits, a number of compounds that provide the antioxidant activities were identified [20]. The latter results open new possibilities of using these indigenous fruits in the nutraceutical and food industries.

Wine is a famous product derived from fruit for which a range of metabolomic studies have been performed [24,25]. Here, combining non-targeted metabolite profiling by UPLC-Orbitrap-MS/MS profiling and sensory analyses revealed commonalities and differences of wines made with different grape cluster types obtained each from three *Vitis vinifera* cv. Pinot noir putative-clones grown in identical field environments [26]. In addition, a set of molecular-feature (metabolite) markers was selected as being associated with these sensorial attributes.

Overall, these 13 articles demonstrate that metabolomics is a powerful and insightful tool to address numerous biological issues that link to fruit biology before or after harvest. The future improvement of metabolite annotation workflows will certainly help to decipher more ambitious questions when metabolomics biomarker discovery broadens our understanding of fruit processes. A greater number of fruit metabolomics studies, especially for tropical species or species other than major crops, will allow for a better depiction of fruit mechanisms that govern development and responses to environmental fluctuations.

To conclude, we would like to thank each of the authors who contributed to this Special Issue on fruit metabolism and metabolomics, the peer reviewers who allowed for improving the quality of submitted manuscripts, and the staff members of the Metabolite Editorial Office for their support.

## Figures and Tables

**Figure 1:**
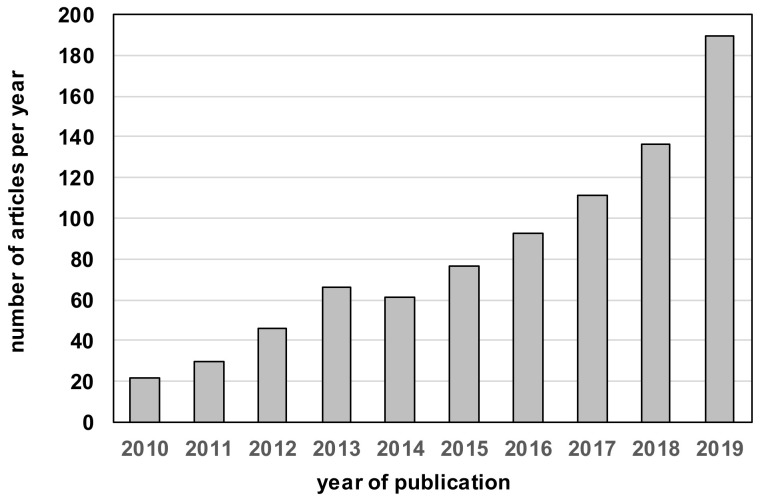
Number of articles published about fruit metabolomics over the past 10 years. Search in Web of Science core collection for TS = (metabolome OR metabolomics *) AND (TS = fruit) NOT (TS=mushroom * OR TS = fruit-fly).

**Figure 2:**
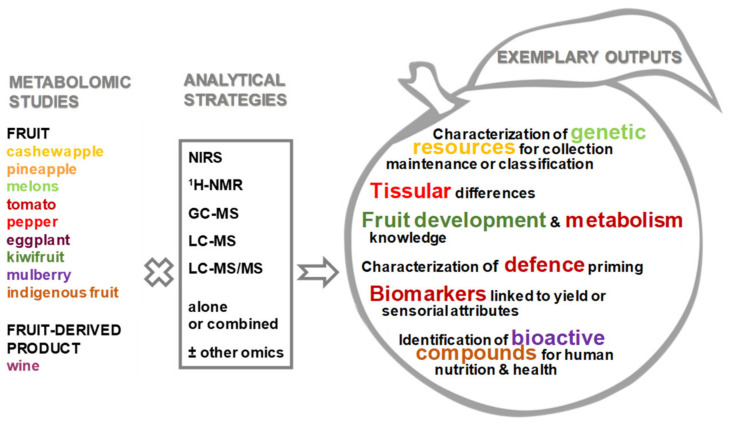
Summary of the works presented in the Special Issue on “Fruit Metabolism and Metabolomics”. GC-MS, gas chromatography coupled to mass spectrometry; ^1^H-NMR, proton nuclear magnetic resonance spectroscopy; LC-MS, liquid chromatography coupled to mass spectrometry; NIRS, near-infrared spectroscopy.
